# Clinical and Radiographic Evaluations of Biodentine™ Pulpotomies in Mature Primary Molars (Stage 2)

**DOI:** 10.5005/jp-journals-10005-1564

**Published:** 2018

**Authors:** Hitaf Nasrallah, Balsam El Noueiri, Charles Pilipili, Fouad Ayoub

**Affiliations:** 1,2 Department of Paediatric Dentistry, Lebanese University, Beirut, Lebanon; 3 Department of Faculté de Médecine et Médecine Dentaire, UC Louvain Brussels, Belgium; 4 Department of Forensic Sciences, Lebanese University, Beirut, Lebanon

**Keywords:** Biodentine, Complete root formation, Primary molars, Pulp canal obliteration, Pulpotomy

## Abstract

**Introduction:**

The preservation of the integrity and health of primary teeth and their supporting tissues is of great importance in maintaining arch length space, mastication, speech, and esthetics. A pulpotomy is a common therapy performed on a primary tooth presenting reversible pulpitis or a traumatic pulp exposure, allowing its conservation on the arch until its loss.

**Aim:**

The study aims to clinically and radiographically evaluate the rates of success and efficacy of Biodentine™ as pulpotomy medicament exclusively on deciduous molars with complete roots formation (stage 2).

**Materials and methods:**

A total number of 75 primary molars in stage 2 of formation were selected to undergo pulpotomy treatment. All teeth were restored with a stainless-steel crown.

The clinical success was evaluated at 1, 3, 6 and 12-month intervals. The radiographic follow-up evaluations were at 6 and 12 months. The resulting data were tabulated and statistically analyzed.

**Results:**

Among the 75 teeth treated with Biodentine™, one tooth revealed abnormal mobility and tenderness to percussion at the end of the 1st month. PLS widening and the bone lesion was not seen in any of the 74 remaining cases. Forty teeth (54.1%) showed pulp canal obliteration (PCO), and none of the cases developed a draining sinus or had increased mobility. At the end of the 1-year follow-up, the clinical and radiographic success rates were 98.7% and 100%, respectively.

**Conclusion:**

Pulpotomies performed with Biodentine™ on stage 2 primary molars were generally very satisfactory and fulfilled all requirements, covering all needs. This innovative bioactive medicament seems to be a “heroic” material. The excellent outcomes of the present study are indicative that Biodentine™ is a promising biomaterial to promote pulp repair after pulpotomy in clinical practice.

**How to cite this article:**

Nasrallah H, El Noueiri B, Pilipili C, Ayoub F. Clinical and Radiographic Evaluations of Biodentine™ Pulpotomies in Mature Primary Molars (Stage 2). Int J Clin Pediatr Dent 2018;11(6):496-504

## INTRODUCTION

The preservation of the integrity and health of primary teeth and their supporting tissues is of great importance in maintaining arch length space, mastication, speech, esthetics and adequateoro-facial development of the child.^1–3^

One of the conservative pulp treatments adopted by pediatric dentists in the pulpotomy.

A pulpotomy is a common therapy performed on a primary tooth when caries removal results in pulp exposure with a normal pulp, reversible pulpitis or after traumatic pulp exposure.^4,5^ This procedure involves the amputation of the coronal pulp leaving the radicular pulp partially or totally vital and healthy.^2,6^

A unique feature of a deciduous tooth is that it goes through three evolutionary stages that influence its reaction to different aggressions. Stage 1 is the period of the immaturity of the root. At this stage, the maturing pulp has a strong dentinogenetic and repair potential. Stage 2 corresponds to the complete maturity period of the tooth. Stage 3 consists of the physiological root resorption of the deciduous tooth, until its loss by the underlying successor.^7,8^

A pulpotomy is an indicated pulp treatment in the three physiological stages of deciduous teeth; it can be performed using different techniques including non-pharmacotherapeutic treatments such as electrosurgery^9^ and laser,^10–12^ or pharmacotherapeutic agents such as formocresol,^13–15^ glutaraldehyde,^16,17^ ferric sulfate,^17,18^ calcium hydroxide,^19–21^ MTA,^22–25^ and CEM cement.^26–29^

Biodentine™ (Septodont, St. Maur-Des-Fosses, France), is the latest bioactive calcium silicate-based cement recently launched in the dental market, characterized by its biocompatibility,^30^ high mechanical properties,^31–33^ remarkable setting time (9–12 minutes),^34^ radiopacity,^35^ superior sealing potential,^36^ enhanced compressive strength and microhardness.^37–40^ Thus, Biodentine™ has bioactive properties^41^ encouraging hard tissue regeneration and increasing cell proliferation and biomineralization.^42^ Indeed it helps to form a reparative dentine as a result of induction of cell differentiation.^39,40,43,44^

A recent study was conducted on stage 3 deciduous molars that underwent pulpotomy with Biodentine™.^45^

The aim of the present study was to investigate the clinical and radiographic success rates of vital pulpotomy with Biodentine™ in human deciduous molars requiring vital pulp therapy and to assess potential differences in clinical and radiological outcomes after pulpotomy on mature deciduous molars (stage 2).

## MATERIALS AND METHODS

### Patient Selection and Study Design

The present study was conducted on a group of healthy children (27 females, 48 males) aged from 5 to 8 years old, presenting 75 stage 2 primary molars (37 first deciduous molars and 38-second deciduous molars) requiring pulpotomy for carious or iatrogenic indications. The patients attended the Department of Pediatric Dentistry at the School of Dentistry of the Lebanese University. They showed good general health and no history of systemic illness. Before the meticulous radiographic and clinical inspection, written informed consent was obtained from the parents or guardians after explaining the full details of the treatment procedure.

The inclusion criteria for the selection of teeth should comprise the following:

- Mechanically or traumatically exposed vital pulp—deep caries
- No history of spontaneous pain or irreversible inflammation
- Normal radiographic findings
- Controlled hemorrhage during the treatment.

Is considered excluded every tooth that shows one or more of the following signs:

- Spontaneous pain
- Radiographic evidence of pulpal or periradicular pathosis,
- Calcification in the pulp chamber
- Pathological mobility
- Presence of fistula or abscess
- Proper hermetic restorations not possible.

The peroperative clinical and radiographic examinations are crucial to confirm the diagnosis of the pulp status; at the time of pulp exposure, the absence of bleeding indicates necrosis, while abundant continuous bleeding after 3–5 minutes of compression would be a sign of pulp inflammation. In these cases, the tooth was excluded from the study.

**Figs 1A to D:**
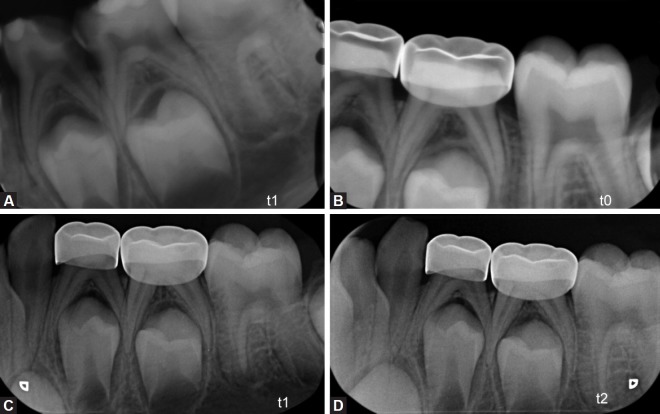
Preoperative and postoperative periapical radiographs of mandibular second primary molar treated with Biodentine™ pulpotomy. (A) Preoperative radiograph; (B) postoperative radiograph taken immediately after Biodentine™ pulpotomy and restored with stainless steel crown; (C) 6-month follow-up periapical radiograph showing pulp canal obliteration of the pulpotomized mandibular second primary molar; (D) follow-up periapical radiograph with pulp canal obliteration

Radiographic evaluation was performed by digital periapical radiographs taken at (t-1): preoperatively during the first examination consultation (t-0): postoperative after pulpotomy and applied stainless steel crown (SSC),(t-1): after 6 months follow-up interval (t-2): after12 months follow-up interval (Fig. 1).

These radiographs were obtained using the Kodak intraoral machine (model 2100) set at 70 Kv, with 0.3 seconds exposure time for the maxillary molars, and 0.2 seconds for mandibular molars.

**Graph 1:**
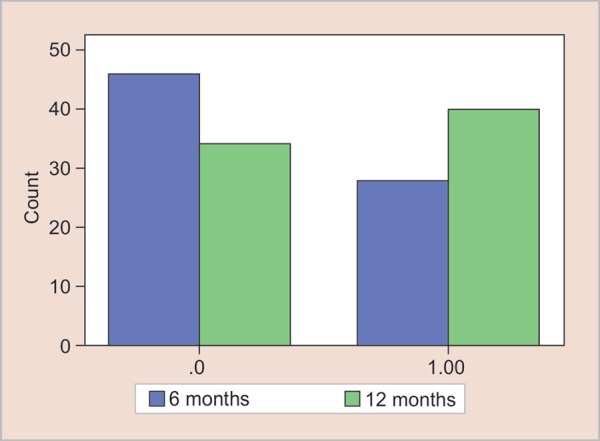
Pulp canal obliteration by months

The pulpotomy procedures were all performed by one operator.

The clinical success was defined by the absence of spontaneous pain, sinus tract, infection, fistula or abnormal mobility. The radiographic success criteria included normal development of successor, presence of a normal periodontal ligament space (PLS), absence of bone lesion and pathologic root resorption.

On clinical and radiographic examinations, if any of these criteria were observed, the treatment was recorded as unsuccessful; however, any radiographic evidence of pulp canal obliteration (PCO) was not regarded as a failure (Graph 1).

### Clinical Procedure

After performing local anesthesia with 5% xilocaine spray and 2% lidocaine injection, the appropriate clamp was selected, and quadrant isolation was performed with a rubber dam. Instruments and burs used in the study were all disinfected and sterilized. Superficial caries was removed with high-speed carbide fissure bur. No disinfection of the coronal cavity was performed according to the standard of care in pulpotomy treatment.

Following pulpal exposure, the superficial coronal pulp was removed with a low-speed carbide round bur no. 2 and the whole coronal pulp was amputated with a sterile sharp spoon excavator.

The pulp chamber was then flushed with 5 cc sterile saline, and a series of sterile cotton pellets saturated with saline were placed in the pulp chamber under light pressure for 2–3 minutes to obtain hemostasis. Once the cotton pellets were removed, Biodentine™ paste was applied over the radicular pulp stumps with an appropriate spatula delivered in the box by the manufacturer. The paste was obtained by mixing premeasured unit dose capsules for 30 seconds at 4200 rpm in a titrator according to the manufacturer's instructions.

After 12 minutes, the Biodentine™ had set, the remaining portion of the pulp chamber and the access cavity were filled with zinc oxide-eugenol cement IRM (Intermediate Restorative Material), the tooth was then restored immediately, with a stainless steel primary molars crown (SSC, 3M Unitek SP, USA) cemented with glass ionomer cement (Ketac-Cem; 3M ESPE, USA).

At the end of the treatment session, the data was recorded on a form for each patient. Clinical parameters were evaluated at 1, 3, 6, and 12-month intervals and the radiographic ones at 6 and 12 months.

In case of loss of the SSC during the follow-up period, the tooth was excluded from the study.

Seventy-five cases were studied to see if there was:

- A difference between clinical parameters (sinus tract, tooth mobility, spontaneous pain, or infection) in the 1st, 3rd, 6th, and 12th months of follow-up.
- A difference between radiological parameters (periodontal ligament widening, bone lesion, radicular pulp canal obliteration or pathologic root resorption and normal development of success or teeth) in the 6th and 12th months of follow-up.
- A difference in the outcome of the pulpotomy at these intervals.

All clinical and radiographic data were tabulated in Tables 1 and 2.

To evaluate the clinical and radiographic variables collected respectively in Tables 1 and 2, a statistical analysis was performed using IBM SPSS for Windows version 20.0 (SPSS, Chicago, IL, USA). The proportions of the variables at 1, 3, 6 and 12-month interval were presented as percentages. The McNemar test was used to compare these proportions between baseline and following months for presence or absence of pathology.

**Table 1:** Clinical follow-up

*Clinical follow-up*																
*Number of cases*	*Status of sinus tract*				*Status of tooth mobility*				*Status of spontaneous pain*				*Status of infection*			
	*1 month*	*3 m*	*6 m*	*12 m*	*1 m*	*3 m*	*6 m*	*12 m*	*1 m*	*3 m*	*6 m*	*12 m*	*1 m*	*3 m*	*6 m*	*12 m*
74	A	A	A	A	A	A	A	A	A	A	A	A	A	A	A	A
1	A	P	—	—	A	P	—	—	A	P	—	—	A	P	—	-—

m: month; A: absence; P: presence; —: not applicable

**Table 2:** Radiographic follow-up

	*Radiographic follow-up*									
	*Periodontal ligament space widening*		*Status of bone lesion*		*Pulp canal obliteration*		*Pathologic root resorption*		*Normal development of successor*	
*Number of cases*	*6 months*	*12 months*	*6 months*	*12 months*	*6 months*	*12 months*	*6 months*	*12 months*	*6 months*	*12 months*
34	A	A	A	A	A	A	A	A	P	P
12	A	A	A	A	A	P	A	A	P	P
28	A	A	A	A	P	P	A	A	P	P
1	—	—	—	—	—	—	—	—	-	—

A: absence; P: presence; —: not detected due to loss

**Table 3:** Pulp canal obliteration comparison in 6th and 12th months

			*PCO 6*		*Total*
			*,00*	*1,00*	
PCO 6	0,00	Count	34	12	46
		% Within PCO 6	73.90%	26.10%	100%
		% of Total	45.90%	16.20%	62.20%
	1,00	Count	0	28	28
		% Within PCO 6	0,0 %	100%	100%
		% of Total	0,0 %	37.80%	37.80%
Total		Count	34	40.00%	74
		% Within PCO 6	45.90%	54.10%	100%
		% of Total	45.90%	54.10%	100%

PCO: pulp canal obliteration

A *p* value of less than 0.05 was considered to show a statistically significant result.

## RESULTS

### Clinical Findings

*Among the 75 teeth treated with Biodentine*™*, one tooth revealed abnormal mobility and tenderness to percussion at the third-month clinical follow-up which resulted subsequently in its radical root canal treatment and dropped from further follow-up. This case was regarded as failure. No other tooth revealed abnormal clinical findings during all the observation period.*

The McNemar test showed that there is no statistically significant difference between the status of a sinus tract, tooth mobility, spontaneous pain, and infection, separately, in the 1st, 3rd, 6th, and 12th months of follow-up (*p* >0.05).

### Radiographic Findings

After the third month follow-up, one case showed an infection, mobility and spontaneous pain and was treated by root canal treatment, that tooth was excluded from the study and not evaluated radiologically in the 6th and 12th months.

The statistical analysis on PLS widening, bone lesion, radicular pulp canal obliteration, pathologic root resorption and development of successors was done for the remaining 74 cases.

### After 6 Months

PLS widening and bone lesion were not seen in any of the cases. The pulp canal obliteration (PCO) was observed in 37.8% of the cases (n = 28). No pathological root resorption was noticed in any of the cases (n = 74). All the successors were progressing normally.

### After 12 Months

At the end ofthe12^th^ month, no bone lesion, PLS widening or pathological root resorption were detected in any of the cases. All the succedaneous teeth were progressing normally. The PCO was seen in 12 more cases than at 6 months (n = 40).

At the 6th month, there were PCO in 28 cases (37.8%), while in 46 cases (62.2%) PCO did not appear. At 12th month, PCO was seen in 40 cases (54.1%). So 12 more cases (16.2 %) had PCO after 6th month (Table 3).

The percentage of success and failure ratio stayed the same (100%) except PCO which increased to 54.1% (n = 40). In other words, PCO was seen in 12 more cases (16.2%) at the end of 12 months (Fig. 1).

## DISCUSSION

The present clinical study was conducted to evaluate the preliminary effects and success rate of Biodentine™ as a pulp-dressing agent during pulpotomy in stage 2 deciduous molars.

Stage 2 is the stage of stability and maturation which extends from complete root formation to clinically detectable resorption, and where the maturing pulp has a strong dentinogenetic and repair potential.^7,8^

To prevent the interference of confounding factors, the current study was conducted under controlled experimental conditions. These conditions included the use of rubber dam, disinfection of the operative materials, gentle irrigation hemostasis and to the complete adaptation of the stainless-steel crown.

Controlling these factors will affirm that the statistical results are exclusively in response to the use of Biodentine™ in teeth pulpotomy.

Pulpotomy treatment of human deciduous molars is a well-known treatment procedure, which is carried out following coronal pulp exposure caused by caries, cavity preparation or trauma.^26^

A considerable number of clinical trials with different techniques, treatment objectives and materials have been performed about pulpotomy in primary teeth.

The outcome obtained was said to be dependent upon the agent or pulp medicament used to treat the radicular pulp stumps.^19,46,47^

The ideal material to be selected for pulpotomy procedure should exhibit favorable characteristics in term of good physical and biological properties. It must be biocompatible, not resorbable and have sealing ability of the remaining pulp tissue, minimal leakage and antibacterial activity.^20^

Formocresol (FC) has been the “gold standard” pulp dressing material using on pulpotomized deciduous molars for the past 70 years.^48^ Formocresol contains formaldehyde, a toxic, potentially carcinogenic/mutagenic compound, and concerns have been raised about its safety and its use in dentistry.^2,19,47^

Different materials have been studied to identify an alternative to FC, and the attention has shifted from preservation to regeneration of residual pulp tissue.^24,46^

Recently, the introduction of new pulp therapy agent like MTA has witnessed new innovations in dentistry.^49^ It showed a high success rate when used as pulpotomy medicament and has gained widespread use a result of its excellent biocompatibility, good sealing ability, low cytotoxicity, and solubility; however, MTA has delayed setting time (about 4 hours), is deemed difficult to manipulate and is expensive to use.^35,50^ This material needs to be in contact with water during the setting reaction. The teeth cannot be left unrestored for this time, so a moistened cotton pellet and an interim restoration were left in the teeth until a subsequent appointment. Hence, the teeth had to be re-entered to remove the cotton pellet, and then they required another restoration.

Ideally, the manufacturer needs to change the physical properties of the material so that the entire procedure can be completed in one appointment.^51^

Biodentine™, one of these innovative materials, was designated for the experimental study to overcome the shortages of the previous ones. This material was shown to possess high biocompatible and mechanical characteristics, acceptable physical properties and favorable resistance to corrosion.^35,49^

The main advantages of Biodentine™ over MTA include its ease of handling, high viscosity, shorter setting time (12 minutes), making it more suitable in clinical use, in addition of containing raw material with a known degree of purity.^52^ This material stimulates the deposition of hydroxyapatite on its surface when exposed to tissue fluids,^53^ presents color stability, nongenotoxic, has low cytotoxicity^3^ and preserves gingival fibroblast viability.^3,52^

Collado-Gonzalez et al. demonstrated that Biodentine™ exhibited better cytocompatibility and bioactivity than MTA Angelus, Theracal LC and IRM on stem cells from human exfoliated primary teeth.^21^ Furthermore, this medicament revealed excellent results for its use clinically. This has prompted its use for pulpotomy in primary teeth.

The use of stainless steel crowns in the present study was aimed to ensure the hermeticity of the cavity and to increase the success rate of pulpotomy.^54^ The most effective long-term restoration of vital pulp therapy in cariously exposed teeth has been shown to be a stainless-steel crown.^5^ This complete-coverage restoration strengthens the weakened crown and protects the underlying pulp against leakage. It has been shown that failure is 7.7 times more likely in a tooth restored with an amalgam than one restored with a preformed metal crown.^55^

At the third month follow-up, one clinical failure had occurred; one tooth showed abnormal mobility and sensitivity to percussion. Presence of status of infection warranted immediate root canal treatment of the designated tooth.

The failure of pulpotomy treatment in this primary molar could be more likely the result of undiagnosed chronic inflammation existing in radicular pulp before pulpotomy rather than due to exposure of radicular pulp to Biodentine™. Gisoure reported similar results on FC pulpotomies and considered that the chronically inflamed radicular pulps were believed by the clinician to be non-inflamed which led to a peroperative clinical misjudgment of the bleeding nature and time.^56^

An inherent problem in treating any exposed pulp is the lack of ability of clinicians to accurately diagnose the true state of the pulp and to predict the pulp's ability to respond to any form of therapy.^49,56^ This is even more difficult in children where the patient's responses to pulp testing procedures are unreliable. The history of previous pulp involvement is an important factor as is the level of penetration of the bacteria associated with caries.^51^

Besides the only rejected case and during the entire observation period of the 12th month, none of the remained cases showed any abnormal clinical findings; none of the pulpotomy cases developed a draining sinus or had increased mobility.

Therefore, at the end of the 1-year follow-up, the clinical success rate was 98.7% and the failure rate was 1.3%. This clinical trial evaluating the performance of Biodentine™ as a pulp-dressing pulpotomy medicament reported a significant degree of pulp healing, excellent sealing ability, and superior biocompatibility of this medicament.

Comparable clinical success rates with Biodentine™ have been reported in the literature even though none of them were conducted on one specific stage of physiological root status.

For Kusum et al., the clinical scores obtained by 25 pulpotomized teeth on healthy patients aged 3–10 years over 9 months follow-up period were 100%.^49^

Cuadros-Fernandez et al. found 100% of clinical success after 12 months of pulpotomy treatment with Biodentine™ performed in patients aged from 4 to 9 years.^57^

At the 12-month follow-up, Rajasekharan et al. obtained with a smaller sample size (25 cases of healthy participants aged 3–8 years) 96% of clinical success withBiodentine™ and 100% with ProRoot WMTA, in 29 cases.^54^

Moreover, by using Biodentine™ as pulp-dressing material, El Meligy et al. obtained 100% of clinical success in all 56 pulpotomized teeth in children aged 4–8 years.^12^

Considering the radiographic evaluation, after the 3rd month, one case was lost due to inflammation, so it could not be evaluated radiologically at 6 and 12 months. The 74 remaining pulpotomized teeth were followed-up radiographically; the present results did not show any PLS widening, bone lesion or pathological root resorption. In addition, there were no obvious changes to the pericoronal sac associated with the underlying developing permanent teeth.

The 100% radiographic success of Biodentine™ is in line with the success rate at 12 months observed in 2016 by El Meligy et al.,^12^ while Rajasekharan et al. reported a slightly lower percentage (96%).^54^

Pulp canal obliteration (PCO) was the most common radiographic findings in pulpotomized molars treated with Biodentine™ (54.1%). This feature is another controversial radiographic outcome with different schools of thought.^2,49,58^ Some classify PCO as a radiographic failure as it showed a deviation from a normal pulp, others argue that PCO or calcific metamorphosis occurs as a result of the extensive activity of odontoblast-like cells which is suggestive of retained vitality, function overtime, and sign of healing and was therefore considered as a success.^19,54^

In the present study, PCO was not considered as a radiographic failure, and was the most commonly observed radiographic outcome.

Out of 74 treated primary molars with Biodentine™, 28 teeth (37.8%) showed pulp canal obliteration at the radiographic evaluation at 6 months. Twelve more pulp obliteration cases were noticed at the 12 month radiographic evaluation (16.2%) which means that the percentage of PCO had increased.

In the few *in vitro* studies available so far, Biodentine™ presented compatibility to dental pulp cells and stimulated the formation of tertiary dentin.^43,46,59^ It had also induced the differentiation of cultured pulp cells into odontoblast-like cells16 and mineralized foci formation, similarly to MTA and calcium hydroxide.^46^

Laurent et al. investigated the capacity of Biodentine™ to induce reparative dentin synthesis by modulating pulp cells to secrete transforming growth factor-beta 1 (TGF-ß1) which main role is the signaling of reparative dentinogenesis and stimulate human dental pulp mineralization.^30^ Histologically, the bioactive tricalcium silicate demonstrated the ability to stimulate dentin regeneration by inducing odontoblast differentiation from pulp progenitor cells.^39^

Shayegan et al. showed that Biodentine used as a pulpotomy material on primary pig teeth has bioactive properties, encourages hard tissue regeneration, and provoke no signs of moderate or severe pulp inflammation response.^41^ Rajasekharan et al. has also reported similar high rates of PCO (48%) for the same follow-up period.^54^ Kusum et al. noticed that PCO was the most common radiographic finding in both MTA (20%) and Biodentine™ (16%).^49^

In the present study, the percentage of PCO (54.1%) on 75 cases is higher than the one obtained by Nasseh et al (accepted 2017) who reported a percentage of 25.7 on 35 stage 3 deciduous molars with reduced healing potential. This difference can be attributed to the high potential of healing of the pulp in stage 2 deciduous molars which can react to physiological stimuli by apposition of reactive dentin. Moreover, the physiology of these teeth is comparable to that of the young permanent ones.^7,8^

In our study, radiographs showed no root resorption, no lesion at the furcation and no periapical radiolucencies. In addition, there were no obvious changes to the pericoronal sac associated with the underlying developing permanent teeth.^51^

The radiographic success rates for Biodentine™ pulpotomies evaluated in this study are in line with those reported at corresponding time periods for Biodentine™ pulpotomies. Cuadros-Fernández et al. in 2016, reported a 95% radiographic success rate after one year compared with a 100% success rate in the current study at 1 year. One molar showed internal resorption and a second exhibited periradicular radiolucency.^57^

For all treated teeth, Biodentine™ showed 100% radiographic success at both the 3- and 6- months follow-ups in a study conducted by El Meligy et al.^12^ Rajasekharan and coworkers, in 2017, reported a radiographic success rate of 94.4% at 18 months.^54^ Kusum et al. have also reported similar high rates of radiographic success (80%) with Biodentine™ for 9 months after a pulpotomy, 4% presented with non-perforated internal resorption radiographically and 8% showed external resorption.^49^

The optimization of Biodentine™ was evident in the current study. This pulp dressing material appears to satisfy the conditions for pulpotomy medicaments, and this may be due to a combination of its bactericidal and germicidal action, excellent sealing ability, biocompatibility, alkalinity, and ability to regenerate hard tissues.

## CONCLUSION

Based on the findings of the present study, and since the treated teeth were retained asymptomatic in the oral cavity, the excellent outcomes obtained are indicative that Biodentine™ is a promising biomaterial to promote pulp repair after pulpotomy procedure on human mature primary molars (stage 2) over 12 months of follow-up. Furthermore, the outcomes of Biodentine™ on stage 3 primary molars were statistically not different than those of stage 2, whereas the percentage of PCO was lower. These findings suggest the potential of Biodentine™ for being used as a pulpotomy medicament in primary teeth.

The clinically favorable response to treatment is possibly due to a combination of factors such as removing all bacteria, the high potential of healing of human stage 2 primary molars, the physical, biologic, handling properties, sealing effect, and the low toxicity of the material used. Biodentine™ seems to have more promising potential to become the medicament of choice for pulpotomy in primary teeth.

Vital amputation with Biodentine™ is a reliable biological method for pulp treatment of primary teeth and could be recommended for the clinical practice. However, further histological studies on larger sample size and a longer observational period should be carried out in pulpotomy in the future.
